# Parkinson’s Disease: Can Targeting Inflammation Be an Effective Neuroprotective Strategy?

**DOI:** 10.3389/fnins.2020.580311

**Published:** 2021-02-25

**Authors:** Vidar Gundersen

**Affiliations:** Section for Movement Disorders, Department of Neurology, Oslo University Hospital, Oslo, Norway

**Keywords:** microglia, alpha-synuclein, T-cells, brain, gut, cervical lymph node

## Abstract

The reason why dopamine neurons die in Parkinson’s disease remains largely unknown. Emerging evidence points to a role for brain inflammation in neurodegeneration. Essential questions are whether brain inflammation happens sufficiently early so that interfering with this process can be expected to slow down neuronal death and whether the contribution from inflammation is large enough so that anti-inflammatory agents can be expected to work. Here I discuss data from human PD studies indicating that brain inflammation is an early event in PD. I also discuss the role of T-lymphocytes and peripheral inflammation for neurodegeneration. I critically discuss the failure of clinical trials targeting inflammation in PD.

## Introduction

Idiopathic Parkinson’s disease (PD) is the most common neurodegenerative movement disorder, afflicting 1% of the population over the age of 60 (de Rijk et al., 1997). The cardinal symptoms of PD (bradykinesia, tremor, rigidity) result from the degeneration of dopamine neurons in the nigrostriatal pathway. Neuronal cell bodies, located in the compact part of the substantia nigra (SNc), send axons to the striatum. The dorsal putamen receives dopaminergic axon terminals from the ventral tier of the SNc (Lynd-Balta and Haber, 1994). These projections are especially involved in motor functions and they are particularly vulnerable to degeneration in PD (Kordower et al., 2013). In PD dopamine neurons in the SNc gradually die. This causes progressive malfunction of the nigro-striatal system and disease progression. The pathological hallmark of PD is loss of dopamine neurons and accumulation of misfolded α-synuclein in Lewy bodies in degenerating neurons in the SNc (Jellinger, 2012). Although Lewy bodies contain about 90 different types of protein, α-synuclein is thought to play a critical role in disease development (Wakabayashi et al., 2013). In the early stages of PD the motor symptoms are reversed by dopamine replacement therapy, but these treatments grow less effective over time. With disease progression motor complications, such as dyskinesia, become treatment limiting. In addition, most PD patients develop non-motor symptoms with disease progression, which have a major impact on quality of life and disability (Schapira et al., 2017). No current treatment slows or stops the neurodegeneration and the progression of PD (Hilker et al., 2005). The main reason why we lack treatment that can stop dopamine neurons from dying, is that the etiology of PD is largely unknown. This is reflected by the fact that the human central nervous system is complex and neuronal function is incompletely understood, making the underlying causes of neurodegeneration difficult to decipher. Our understanding of pathogenic mechanisms in idiopathic PD is mainly based on results from experimental animal PD models. So far data from these studies have not successfully been translated into effective neuroprotective treatment. Recent data from human studies suggest that brain inflammation is an important contributor to neurodegeneration in PD. In this review I will discuss the role of inflammation in neurodegeneration and whether inflammation could be a potential therapeutic target in idiopathic PD. I will focus on human idiopathic PD. There are several reviews discussing inflammation in experimental toxic and genetic PD models; for a recent excellent review bridging human and experimental PD (see Schonhoff et al., 2020).

## Parkinson’s Disease and Neurodegeneration

Several mechanisms are proposed to underlie the progressive death of dopamine neurons in PD, but it remains unclear which mechanism that are the main driver of the disease process. Both oxidative stress, caused by mitochondrial dysfunction and enzymatic metabolism of dopamine by monoamine oxidase (MAO) (Burbulla et al., 2017), as well as impaired protein degradation and brain inflammation (Olanow, 2007) may contribute to neurodegeneration. Nigral dopamine neurons are thought to be especially vulnerable because they have large arborizations and a vast number of synaptic nerve terminals, putting a large metabolic stress on these neurons (Diederich et al., 2019).

The neurodegenerative process probably starts many years before the start of motor symptoms. Postmortem analyses have shown that at the time of diagnosis about 30% of dopamine neuronal cell bodies in the SNc (Fearnley and Lees, 1990; Greffard et al., 2006; Kordower et al., 2013) and 50% of dopamine axon terminals in the dorsal putamen are lost (Scherman et al., 1989; Kordower et al., 2013). Thereafter, the degeneration is quite rapid. By 4 years after diagnosis there is an almost total loss of dopamine axon terminals, whereas the degeneration of neuronal cell bodies is a bit slower. However, by 5 years the majority of cell bodies is lost (Kordower et al., 2013). This means that if dopamine neurons are to be rescued, neuroprotective treatments must start as soon as possible. After 4–5 years from the time of diagnosis there will probably be no neurons left to rescue. It should be mentioned that after many years of disease, Kordower et al. (2013) reported that there still was a proportion of dopamine neurons left in the ventral tier of the SNc, which seemed to be resistant to further degeneration. Also, there was a higher loss of neurons positive for dopamine markers than of the total pool of melanized nigra neurons, but there was a marked early loss also of the latter pool. This could mean that the surviving melanized neurons are dopamine producing neurons, which have lost their dopamine phenotype as a consequence of disease progression. If neuroprotective treatment will restore their dopaminergic phenotype is not known. If this is the case, protective treatment could have some effect also if started at later stages in the disease process. However, the majority of SNc neurons, both melanized and those with a dopaminergic phenotype, are lost early in the disease, stressing the need of starting neuroprotective treatments as early as possible.

## Inflammation Is an Early and Substantial Event in Parkinson’s Disease

The question is therefore whether there is any evidence that inflammatory processes start early enough so that treatments targeting inflammatory cascades can be expected to have neuroprotective effects. Important in this respect is also whether the contribution from inflammation is so large that inhibiting this process will slow or halt the degeneration of dopamine neurons.

### Brain Inflammation and Microglia Activation

Pivotal to the inflammatory cascades putatively leading to death of dopamine neurons in PD is microglia activation. Under physiological conditions microglia are “resting” or surveillant. In the rodent brain it has been shown that they are finely ramified (Sogn et al., 2013) and quite evenly distributed throughout the brain parenchyma, where they continually survey their microenvironment with mobile processes and protrusions (Nimmerjahn et al., 2005). Microglia can move around because they contain high levels of the contractile protein actin and by doing so, at a given time, their tiny delicate processes contact about 3% of excitatory synapses in the brain (Sogn et al., 2013). As a part of the innate immune system, microglia are able to sense neuron derived danger-associated molecular patterns resulting in microglia activation (Wolf et al., 2017). Upon activation microglia become larger and thicker, showing a spheric shape (Nimmerjahn et al., 2005). Activated microglia exists in several phenotypes, which probably represent a continuum from neuroprotective to neurotoxic phenotypes (Ransohoff, 2016a). When microglia are protective they usually take on phagocytotic properties, eating cellular debris and they secrete anti-inflammatory substances, e.g., interleukin-(IL) 10 and transforming growth factor β (TGF-β) (Le et al., 2016). Toxic microglia secrete pro-inflammatory cytokines, such as IL-1β, IL-6, interferon-γ (INF-γ), and tumor necrosis factor-α (TNF-α) (Qin et al., 2016), and they produce pro-inflammatory enzymes, such as nitrogen monoxide synthase and release nitrogen monoxide (Hunot et al., 1996).

#### α-Synuclein Can Activate Microglia

**FIGURE 1:**
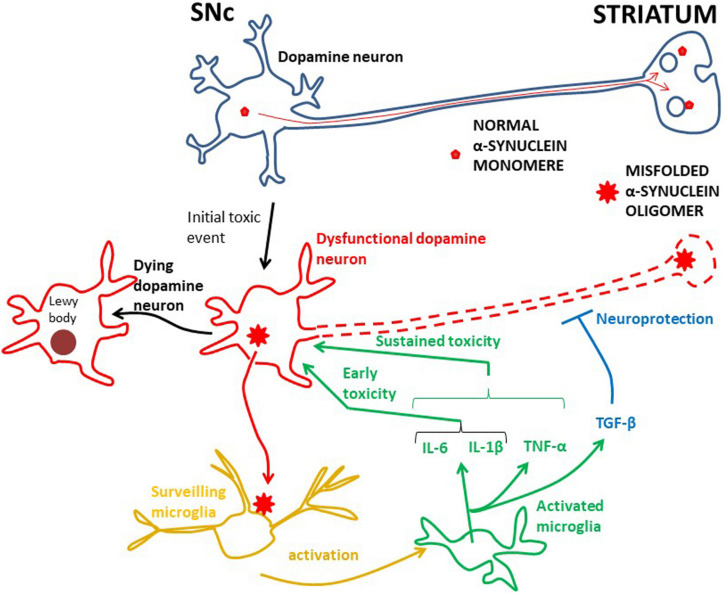
The root cause of PD is unknown, but inflammation is probably an early damaging event in PD. Probably genetic and/or environmental factors contribute to starting the α-synuclein pathology in dopamine neurons. In physiology α-synuclein (“normal α-synuclein”) is transported from the SNc along axons to nerve terminals in the striatum where it is important for synaptic transmission. In dysfunctional dopamine neurons α-synuclein is misfolded into oligomers (“misfolded α-synuclein oligomer”), which can be secreted into the interstitium. This aberrant α-synuclein can activate microglia, in turn starting an inflammatory process through in particular IL-6 and IL-1β. Whether TNF-α works at early PD stages has not been investigated, but TNF-α is involved in sustained brain inflammation. INF-γ may also be part of the sustained inflammatory response, but the data for this are less robust. These cytokines can potentiate the toxic effect on dopamine neurons and the neurodegenerative process, ultimately leading to aggregation of α-synuclein in Lewy bodies and neuronal death. The anti-inflammatory TGF-β, and perhaps IL-10, are probably secreted to counteract the toxic effects of the pro-inflammatory cytokines. However, in PD, the toxic effects seem to prevail. The effect of activated microglia and cytokine release on the death of dopamine neurons is not known for the human brain. A cue may be achieved by studying human iPSC microglia–neuron co-cultures.

In PD, as in other neurodegenerative diseases, there is a wealth of data suggesting that toxic microglia dominate, killing dopamine neurons (Ransohoff, 2016b). Microglia can be activated by a variety of factors including neurotransmitters, pro-inflammatory cytokines and bacterial toxins, such as lipopolysaccharide (LPS) (Gao et al., 2008). Mounting evidence indicates that α-synuclein, the main protein component in Lewy bodies in dying dopamine cells, is essential for activating microglia (Figure 1; Austin et al., 2006; Allen Reish and Standaert, 2015; Sanchez-Guajardo et al., 2015). In experimental animal models of PD misfolded α-synuclein is released from injured neurons into the extracellular fluid. Here it activates microglia through various surface receptors and induces a pro-inflammatory response with production and secretion of e.g.,IL-1β, IL-6, INF-γ, and TNF-α (El-Agnaf et al., 2003; Su et al., 2008, 2009; Chung et al., 2009; Alvarez-Erviti et al., 2011; Béraud et al., 2013; Kim et al., 2013; Roodveldt et al., 2013; Cebrián et al., 2014). This will ultimately damage dopamine neurons (Mount et al., 2007; Cebrián et al., 2014; Main et al., 2016; Zhang et al., 2017). Importantly, also in PD patients there is ample evidence that aberrant α-synuclein is secreted from neurons into the extracellular fluid, where it can be detected in the cerebrospinal fluid (Tokuda et al., 2010; Wang et al., 2012; Majbour et al., 2016).

Probably as an initial event in PD pathogenesis α-synuclein can undergo conformational change to a β-sheet-rich structure, which polymerizes to form toxic soluble oligomers and longer fibrils. These ultimately precipitate as intracellular insoluble plaques in Lewy bodies (Figure 1). Enriched in Lewy bodies are phosphorylated α-synuclein at S129 (Spillantini et al., 1998), which is considered to be a hallmark of pathological α-synuclein (Fujiwara et al., 2002). α-synuclein assembled into such oligomers and fibrils is thought to be toxic to neurons, leading to neurodegeneration (Conway et al., 2001; Luk et al., 2012; Froula et al., 2019), but whether this actually happens through activation of microglia is incompletely understood. In this context, it is noteworthy that in microglia-neuron co-cultures *in vitro* α-synuclein did not produce any neurotoxic effect in the absence of microglia (Zhang et al., 2005), suggesting that α-synuclein may trigger neurotoxicity through inducing microglia activation. Several other *in vitro* studies have shown that α-synuclein plays a key role in microglia activation (Thomas et al., 2007; Klegeris et al., 2008) and that α-synuclein induced activation of microglia causes death of dopamine neurons (Kirik et al., 2002; Eslamboli et al., 2007). Likewise, it has recently been shown that fibrillar α-synuclein was toxic to neurons only though through microglia (Acuña et al., 2019).

### Early Microglia Activation in PD

Whether activation of pro-inflammatory microglia is the cause or just a consequence of damaged dopamine neurons in the PD brain is unclear. However, what is clear is that animal PD models and human PD studies have shown that inflammation is an early event in the progression of PD.

Experimental PD models have demonstrated that activation of microglia precedes dopamine neuron degeneration (Zhang et al., 2005; Su et al., 2008, 2009; Marinova-Mutafchieva et al., 2009; Sanchez-Guajardo et al., 2010). Indeed, α-synuclein overexpression in rats induces alterations in dopamine neuron properties and impairs motor behavior long before dopamine neuron degeneration, which is dependent on microglia activation (Krashia et al., 2019).

However, PD is an exclusively human disease. Although damaging the dopamine nigrostriatal pathway in animals by toxins or genetically gives Parkinson-like behavioral symptoms, such models do not recapitulate the human disease. Thus, they cannot be used as reliable models in which to test neuroprotective strategies. This is supported by the fact that anti-inflammatory agents, which have been found to be neuroprotective in animal Parkinson models, do not show neuroprotective effects when tested in human patients with PD. This is the case for minocycline, a tetracycline with anti-inflammatory properties, which is neuroprotective in 1-methyl-4-phenyl-1,2,3,6-tetrahydropyridine (MPTP) models of PD (Du et al., 2001; Wu et al., 2002), but not in human PD (NINDS Net-PD Investigators, 2008; Parashos et al., 2014). The same is true for agents inhibiting peroxisome proliferator-activated receptors (PPARs), which can induce microglia activation (Agarwal et al., 2017). These agents have shown neuroprotection in animal PD models (Carta et al., 2011; Pisanu et al., 2014), but not in patients with PD (NINDS Exploratory Trials in Parkinson Disease (NET-PD) FS-ZONE Investigators, 2015). Thus, in the discussion below I will focus on data from human studies. The failure of these anti-inflammatory therapeutic approaches in human PD could be related to the complexity of the central nervous system in humans compared to animals and the multifactorial nature of neurodegeneration in PD. Of importance could also be a possible weak potency of the drugs in question and challenges related to clinical trial design, such as inclusion of PD patients with advanced disease (as discussed below).

#### Early Microglia Activation and Cytokines in Brains of PD Patients

The most compelling human evidence indicating that early microglia activation plays an essential role in dopamine neuron pathology in PD stems from postmortem analysis of healthy embryonic dopamine neurons grafted into the striatum of PD patients (Olanow et al., 2019). In the grafts there were signs of inflammation with invasion of activated microglia several years before the grafted dopamine neurons accumulated α-synuclein. This suggests that microglia activation could contribute to the development of α-synuclein pathology in the implanted dopamine neurons. Indeed, increased inflammation in the PD brain has been linked to accumulation of pathological α-synuclein (Dzamko et al., 2017).

In line with this are studies showing microglia activation in incidental PD, a prodromal PD form, where a-synuclein accumulation is found in dopamine neurons at autopsies, but clinically these individuals have not yet developed PD (Doorn et al., 2014). Also in patients with prodromal idiopathic rapid-eye-movement sleep behavior disorder, who did not show any sign of parkinsonism or cognitive impairment, there is evidence of brain inflammation on translocator protein positron emission tomography imaging (TSPO PET) in the SNc (Stokholm et al., 2017). In addition, TSPO PET of patients with Parkinson’s disease with disease duration of less than 2 years showed inflammation in the midbrain, including SNc (Ouchi et al., 2005; Iannaccone et al., 2013). However, there is inconsistency in the TSPO PET results, because one study showed evidence of inflammation in several brain regions other than the SNc (Gerhard et al., 2006), and another study showed no signs of brain inflammation at all (Varnäs et al., 2019). This somewhat questions the reliability of TSPO PET to detect brain inflammation in PD. On the other hand, transcriptome analysis of SN tissue from incidental PD has shown that there is activation of immune responses, as well as evidence of axonal dysfunction and deregulation of synapses (Dijkstra et al., 2015). This points to an early involvement of inflammation, together with synaptic impairment in PD. Whether inflammation contributes to such a “dying-back” neurodegeneration (i.e., that the degeneration starts in axon terminals in the putamen and spreads retrogradely to the SNc (Chu et al., 2012) in PD is not settled.

Despite some inconsistency, altogether, these morphological/imaging/transcriptome studies indicate early involvement of activated microglia in PD (Figure 1). To identify which cytokines these early activated microglia secrete one must turn to biochemical analysis of cerebrospinal fluid (CSF), which can be sampled at all stages of PD. Two CSF studies of PD patients at early stages (disease duration 3 years or less) have been performed. First, IL-1β and IL-6 were shown to be increased in PD compared to control subjects (Blum-Degen et al., 1995). The authors could not detect the cytokines in the blood, indicating that the cytokines have a central origin. Later the same group found that the IL-6 concentration in the CSF was increased in *de novo* Parkinson patients compared to controls and that the concentration was inversely correlated to disease severity (Müller et al., 1998). This suggests that microglia secrete, in particular, IL-6 at an early Parkinson stage, but probably also IL-1β (Figure 1).

#### Sustained Microglia Activation and Cytokines in Brains of PD Patients

There are several human studies showing sustained brain inflammation in advanced PD cases. First, postmortem studies have shown that activated microglia are present in both the SN and the putamen (McGeer et al., 1988a, b; Banati et al., 1998). By immunohistochemistry it has been shown that these cells were enriched with TNF-α (Imamura et al., 2003). In addition, individuals developing PD caused by intoxication with MPTP showed the presence of activated microglia at postmortem examination many years after the insult (Langston et al., 1999), suggesting that a single toxic event may lead to long lasting and continuous brain inflammation. Second, biochemical methods have detected increased brain tissue levels of TNF-α, IL-1β, IL-6, IFN-γ, and TGF-β in the postmortem PD striatum compared to controls (Mogi et al., 1994a, b, 1996a, 1995, 2007). Third, in the CSF there are higher levels of TNF-α (Mogi et al., 1994b; Delgado-Alvarado et al., 2017; Schröder et al., 2018; Iwaoka et al., 2020), IL-1β (Mogi et al., 1996b; Yu et al., 2014; Iwaoka et al., 2020), IL-6 (Mogi et al., 1996b; Schröder et al., 2018; Lian et al., 2019), and TGF-β (Mogi et al., 1995) in PD vs. controls. It should be mentioned that several groups could not find any significant difference in some of the cytokines between PD and controls. This was true for IL-6 (Lindqvist et al., 2013; Yu et al., 2014; Delgado-Alvarado et al., 2017; Starhof et al., 2018; Iwaoka et al., 2020), IFN-γ (Starhof et al., 2018; Schröder et al., 2018; Iwaoka et al., 2020), TNF-α (Lindqvist et al., 2013; Yu et al., 2014; Starhof et al., 2018), as well as for IL-1β (Starhof et al., 2018; Lian et al., 2019), IL-10 (Starhof et al., 2018; Schröder et al., 2018), and TGF-β (Starhof et al., 2018). Moreover, Iwaoka et al. (2020) could not detect IL-1β and IFN-γ in the CSF of PD patients. Karpenko et al. (2018) found detectable levels of TNF-α, IL-1β, IL-6, and IL-10 in PD CSF, but they did not compare with a control group. IFN-γ has also been detected in the CSF at lower concentrations in PD compared to controls (Yu et al., 2014).

Taken together, although information about early cytokine production in PD is somewhat scarce, it supports the idea that microglia are activated early in the disease process and suggests that they produce pro-inflammatory cytokines, especially IL-6 and IL-1β (Figure 1). It has not yet been investigated whether TNF-α and IFN-γ are also produced at early stages, but along with IL-1β and IL-6, TNF-α probably contributes to persistent neuroinflammation, while the data are less robust for IFN-γ (Figure 1). The cytokine studies suggest that also the anti-inflammatory cytokine TGF-β could be part of the microglial phenotype in progressive PD (Figure 1), but whether IL-10 is significantly produced in the CNS during the course of PD is not so clear. These considerations are in line with a meta-analysis of cytokine CSF-concentrations in advanced PD patients, showing that IL-6, IL-1β, and TGF-β were increased compared to controls (Chen et al., 2018). The authors could not detect any significant difference for TNF-α, but there was a much higher heterogeneity in this data set, as well as a lower number of analyses than for IL-6, IL-1β, and TGF-β.

Thus, microglia may produce a mix of pro- and anti-inflammatory cytokines, reflecting the complex nature of the inflammatory cascades taking place in the parkinsonian brain (Joers et al., 2017). TGF-β could be secreted to counteract the toxic effects of IL-6, IL-1β, and TNF-α, but the significance of this is not known. To my knowledge the potential toxic effect of activated human microglia on human dopamine neurons has not been studied. This may be done by investigating the survival of dopaminergic neurons derived from human induced pluripotent stem cells (hiPSC) (Swistowski et al., 2010) in a mixed culture with mature hiPSC-derived microglia (Muffat et al., 2016). Such human iPSC microglia–neuron co-cultures could be made from patients with idiopathic PD and compared with those from healthy controls.

The cytokines observed in brain tissue and CSF are most likely produced by microglia. However, it should be mentioned that reactive astrocytes are also present in the PD brain. These have been shown to produce certain pro-inflammatory cytokines, although it is generally thought that astrocytes rather produce and secrete neuroprotective factors, such as glial cell line-derived neurotrophic factor (GDNF), brain-derived neurotrophic factor (BDNF) and mesencephalic astrocyte-derived neurotrophic factor (MANF) (Pöyhönen et al., 2019). Recent biomarker studies have examined the levels of inflammation related proteins in the CSF of PD patients. Chitinase-3-like protein 1 (CHI3L1)/YKL-40 is such an inflammatory associated protein, which is secreted from, in particular, reactive astrocytes in the inflamed brain (Bonneh-Barkay et al., 2010). With respect to YKL-40 and PD, results from CSF studies are conflicting; some studies found the same concentration in PD patients as in controls (Magdalinou et al., 2015; Wennström et al., 2015; Hall et al., 2016), whereas others found YKL-40 to be lower in PD patients compared to controls (Olsson et al., 2013; Hall et al., 2018). However, YKL-40 was associated with disease progression (Hall et al., 2016), supporting the idea that reactive astrocytes are involved in brain inflammation and neurodegeneration in PD. Another inflammation related protein thought to be involved in PD is the soluble part of the triggering receptor expressed on myeloid cells 2 (sTREM2), which comprises the extracellular domain of the full length TREM2 receptor protein. TREM2 seems to be located in microglia, at least in rodents (Frank et al., 2008), but if human microglia produce TREM2 is unclear (Fahrenhold et al., 2018). In experimental animals activation of TREM2 stimulates a phogocytotic phenotype of microglia and dampens cytokine production, but sTREM2 does the opposite; it activates microglia to secrete pro-inflammatory cytokines (Zhong et al., 2017). sTREM2 is increased in the CSF from PD patients compared to controls (Wilson et al., 2020). In the latter study sTREM2 was correlated with Tau, meaning that it may signal at the same time ongoing processes of inflammation and neurodegeneration.

**Box 1.**

Activation of the adaptive immune system requires recognition of foreign antigens. These are recognized by a diversity of T cell receptors (TCR), enabling them to recognize any protein antigen. TCRs are expressed on the surface of Tlymphocytes. They disclose the presence of protein antigens by binding short peptides, which are cleavage products of the protein antigen. Such peptides are presented on the surface of cells bound to class I or class II molecules of the major histocompatibility complex (MHC). MHC class I is present on all nucleated cells, whereas MHC class II is located on professional antigen presenting cells (APCs), such as microglia. Peptides presented on MHC class I are recognised by T-cell receptors on cytotoxic CD8+ T cells and those on MHC class II are sensed by Tcell receptors on CD4+ T helper cells. Class I molecules usually presents endogenous derived peptides, often from proteasomal breakdown of for instance viral proteins. This will trigger a cytotoxic response of CD8+ T-cells by release of cytotoxic granules comprising perforin and granzymes. Perforin forms a pore in the membrane of the target cell, allowing the granzymes to enter the target cell. As granzymes are proteases they induce apoptosis of the target cell.

On class II MHC molecules the peptides are usually derived from extracellular antigens, which have been phagocytosed by the antigen presenting cell. TCR recognition of such peptide/MHCII complexes primes naīve CD4+ Tcells to differentiate into specific subtypes depending on e.g. the cytokine repertoire secreted by the APC. The release of IL-12/IFN-γ results in T-helper 1 (Th-1) cells, while the release of IL-4 generates T-helper 2 (Th-2) cells. The concentrations of TGF-β/IL-6 are involved in the differentiation of T-helper 17 (Th-17) and regulatory T cells (Tregs). The effector functions of CD4+ T-cells are mediated by cytokines secreted by the differentiated cells. The typical Th1 cytokine profile comprises IFN-γ and TNF-α and the Th-17 profile consists of IL17 and IFN-γ. These cytokines can be toxic to other cells, such as neurons in the brain. They trigger activation of microglia in the innate immune system, Blymphocytes and CD8+ T-cells, as well as regulate Tregs in the suppression of immune reactions. The functional roles of CD4+ and CD8+ -cells in the brain are incompletely understood.

#### Cytokines in the Blood of PD Patients

Several studies have measured cytokine concentrations in the blood. Two studies have investigated blood cytokine levels in early PD (within 2 years from diagnosis). The first one showed that TNF-α, IL-1β, and IL-10 were higher in PD than in controls, but they could not find any difference for INF-γ (Williams-Gray et al., 2016). Later, Kim et al. (2018) found that the levels of IL-1β were higher in PD compared to controls, but this was not the case for IL-6, IL-10, and TNF-α. In advanced PD several studies have measured cytokine levels. A recent meta-analysis showed that blood concentrations of IL-1β, IL-6, IL-10, and TNF-α are increased in patients with PD (Qin et al., 2016). Also, IFN-γ seems to be increased in the blood of advanced PD patients compared to controls (Mount et al., 2007). Thus, it seems as if especially IL-1β is present at high levels in the blood of PD patients from the start of motor symptoms. In addition, several cytokines, including IL-1β, IL-6, IL-10, TNF-α, and probably IFN-γ could be part of an ongoing systemic inflammation along disease progression in in PD.

It is known that subpopulations of CD4+ T-helper lymphocytes secrete cytokines (Box 1). These cells can be differentiated into pro-inflammatory cells, such as T helper 1 (Th 1) and T helper 17 (Th17), and anti-inflammatory phenotypes, such as T helper 2 (Th2) and the T regulatory (Treg) cells. Pro-inflammatory Th1 helper cells secrete IFN-γ and TNF-α, suggesting that these cells may have contributed to the increased blood levels of these cytokines in PD (Kustrimovic et al., 2018). Also, IL-17, which is typically secreted from Th-17 cells, are found to be elevated in the blood of PD patients (Sommer et al., 2018). The IL-10 finding may be mediated by Tregs, while the elevated levels of IL-1β and IL-6 most likely reflect production by peripheral macrophages. Together, the increased blood levels of cytokines suggest a peripheral site of injury with activation of peripheral immune cells during the course of PD (see discussion below). However, a reason for the elevation in blood cytokines could also be a “wash out” from the brain via the glymphatic system (Iliff et al., 2012). Moreover, it has also been observed decreased plasma concentrations of several cytokines in PD; e.g., IL-6, IL-1β, IL-10, TNF-α, IFN-γ, and IL-17 (Hasegawa et al., 2000; Rocha et al., 2018), indicating rather an impaired cytokine production in advanced PD.

### Involvement of Adaptive Immunity and Activation of T-Cells in PD

**FIGURE 2:**
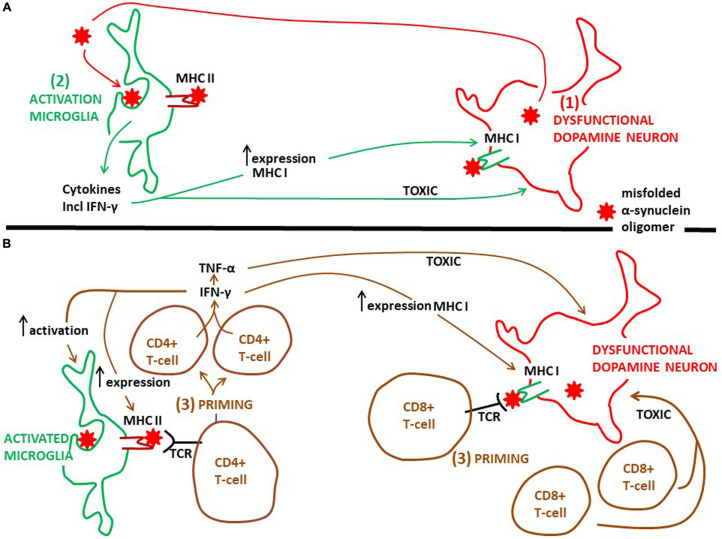
Proposed time course and the action of microglia and CD4+ and CD8+ T-cells on degeneration of dopamine neurons in PD. **(A)** Probably as an initial event in PD pathogenesis dysfunctional dopamine neurons secrete misfolded α-synuclein oligomers, which can activate microglia (see Figure 1). Next, activated microglia phagocytose this α-synuclein and present aberrant α-synuclein peptides via MHC class II molecules to CD4+ T-cells. It is well established that activated microglia present antigens on MHC II, but which type of antigens that are presented in the human PD brain is not known. Activated microglia release pro-inflammatory cytokines, including INF-γ (see Figure 1). The cytokines are toxic to dopamine neurons, further augmenting the pathology. INF-γ can increase the expression of MHC class II on microglia and MHC class I on dopamine neurons. **(B)** Priming of CD4+ T-cells, as well as of CD8+ T-cells, happens after microglia activation, but exactly when in the time course of PD progression is not known. Whether priming of CD4+ and CD8+ T-cells occurs within or outside (see Figure 3) the PD brain is not clarified. INF-γ, which can be secreted also from primed CD4+ T-cells, sustains the activated phenotype of microglia and further increases the expression of MHC class II on microglia and MHC class I on dopamine neurons. This will stimulate the priming process. Besides INF-γ, primed CD4+ T-cells may secrete TNF-α and primed CD8+ T-cells may release secretory granules, both of which may be toxic to neurons. But if these T-cell mediated toxicities take place in the brain of PD patients is not known. TCR, T-cell receptor.

As discussed above the innate immune system seems to contribute to neurodegeneration in PD. Activation of microglia, which can sense α-synuclein and secrete pro-inflammatory cytokines, will promote neuronal pathologies (Figure 1). This is supported by genome wide association studies (GWAS), which have shown that pathways involved in inflammation and regulation of cytokine production are related to PD (Durrenberger et al., 2012; Holmans et al., 2013). Moreover, a large number of the genes associated with PD risk alleles are expressed by microglia (Gosselin et al., 2017). Thus, microglia may form a link between innate and adaptive immunity in PD (Figure 2).

In the healthy human brain postmortem immunolocalization studies have demonstrated that major histocompatibility complex (MHC) class II molecules are exclusively present on “resting” microglial cells (Hayes et al., 1987; Lowe et al., 1989). This constitutive expression is low compared to the density of MHC class II on activated microglia (Hayes et al., 1987). Postmortem analyses of PD brains have shown that MCH class II is highly expressed on the surface of activated microglia. First, McGeer et al. (1988a, b) demonstrated that human leukocyte antigen (HLA)-DR-positive reactive microglia were present in the SN in PD to a much greater extent than in non-neurological cases. Then, it was shown that the number of reactive microglia positive for MHC class II (using an antibody recognizing HLA-DP, -DQ, and -DR) increased as the dopamine neuron degeneration developed (Imamura et al., 2003). Adding to this is that genetic data from idiopathic PD link MHC class II to the disease. GWAS have identified an association with several MHC class II genes, e.g., HLA-DRB1, HLA-DRB5, HLA-DRA, HLA-DQA1, and HLA-DQB1, as well as the MHC class I molecules HLA-B and HLA-C (Hamza et al., 2010; Wissemann et al., 2013; Pierce and Coetzee, 2017).

Thus, by expressing a variety of MCH class II molecules microglia can act as antigen presenting cells (Figure 2; Lowe et al., 1989). This microglia property may play an important part in the pathogenesis of PD. It is well known that MHC class II presents antigens to CD4+ T-cells (Figure 2; Box 1). In experimental PD models α-synuclein triggers generation of MHC class II on microglia, which is essential for a complex interaction with infiltrating CD4+ T-cells. In this interaction cytokines are produced (e.g., IFN-γ and TNF-α), ultimately resulting in dopamine neuronal degeneration (Harms et al., 2013). The identity of the peptides presented by microglial MHC class II has not been clarified. In addition, in the human PD brain the evidence that microglia present antigens to CD4+ T cells is lacking. However, T-cells isolated from the blood of PD patients are reactive toward epitopes derived from α-synuclein (Sulzer et al., 2017). The authors found that the α-synuclein epitopes comprised those containing phosphorylation at S129, implying that the T-cells recognized aberrant α-synuclein. Moreover, the T-cells from patients with PD that responded were mostly CD4+ T-cells and a few CD8+ cytotoxic T cells interacting with MHC class II and MHC class I, respectively. α-synuclein peptides that bound to MHC class II with high affinity were encoded by HLADRB1 and DRB5 (Sulzer et al., 2017). These HLA variants are the same as those noted to be associated with PD by GWAS (see above). This is intriguing because increased CD4+ T cell infiltration, along with CD8+ T-cells, has been found in the postmortem SN of PD patients compared to healthy controls (Brochard et al., 2009). In addition, the latter authors gave experimental evidence using an animal PD model that it was the CD4+, and not the CD8+ cells, which contributed to loss of dopamine neurons. Thus, it seems as if, in particular, cells expressing MHC class II may present peptides of aberrant α-synuclein to CD4+ T-cells, although this may also be the case for MHC class I and CD8+ T-cells (Figure 2). These interactions may generate autoreactive T-lymphocytes, recognizing disease-altered self-proteins as foreign antigens, suggesting that Parkinson’s disease could be an autoimmune disease (Sulzer et al., 2017). Interestingly, as Tregs are important for the maintenance of immunological self-tolerance, dysfunctional Tregs may contribute to development of autoimmune disorders (Sakaguchi et al., 2020). Indeed, it has been observed that Tregs from patients with PD have an impaired ability to suppress effector T cells compared to healthy controls (Saunders et al., 2012). Hence, dysfunctional Tregs may be involved in the escape from tolerance underlying the development of autoreactive T-cells observed by Sulzer et al. (2017).

**FIGURE 3:**
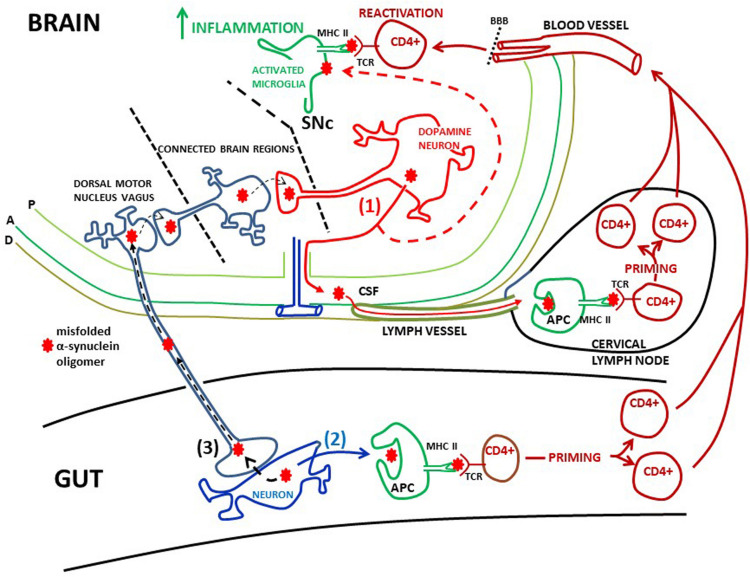
Possible peripheral sites of α-synuclein induced activation of T-cells. (1) Misfolded α-synuclein oligomers are secreted from dopamine neurons in the SNc into the intersitium. From here α-synuclein can flow via the glymphathic system to the CSF and into meningeal lymph vessels and reach cervical lymph nodes (red filled arrows). Here α-synuclein may be phagocytosed by antigen presenting cells (APC) and presented to CD4+ T-cells via MHC class II and T-cell receptors (TCRs), leading to priming of the T-cells. These T-cells can enter the circulation, cross the blood brain barrier (BBB) and patrol the brain parenchyma. Extracellular α-synuclein, released from dopamine neurons, will also activate microglia (red dotted arrow). Microglia will expose α-synuclein on MHC class II. When patrolling T-cells meet antigen presenting microglia they may be reactivated, which in turn may lead to increased brain inflammation. Meninges: P, pia mater; A, arachnoidea; D, dura mater; (2) CD4+ T-cells could be primed in the gut. Local pathology, such as inflammation, may cause α-synuclein to misfold and aggregate in gut neurons. This misfolded α-synuclein could be secreted (black filled arrow) and end up in MHC class II on APCs. This will prime CD + T-cells, which then may gain access to the brain via the circulation in the same manner as T-cells primed in cervical lymph nodes. The finding of α-synuclein reactive T-cells in the blood of PD patients is consistent with scenarios (1) and (2). (3) Misfolded α-synuclein can be trans-synaptically taken up by the vagus nerve and retrogradely transported to the dorsal motor nucleus of the vagus (black dotted arrows). As this nucleus is not directly interconnected with the SNc, α-synuclein can reach SNc through trans-synaptical and retrograde spread via several brain regions. Thus, misfolded α-synuclein from gut neurons may cause templating in dopamine neurons, leading to aggregation of α-synuclein in dysfunctional neurons, inflammation, and ultimately neuronal death.

Recently, evidence was given that α-synuclein specific T-cell responses are present very early in the course of PD, and perhaps before motor symptoms are evident (Lindestam Arlehamn et al., 2020). This underscores the idea that inflammatory cascades are triggered at an early disease stage. However, when the priming of these α-synuclein specific T-cell takes place compared to the activation of microglia in the PD brain is not clear. Moreover, there are no exact data on where T-cells are activated over the course of PD. The mechanisms whereby activated T-cells may injure dopamine neurons the brains of PD patients are also rather obscure. The α-synuclein reactive T-cells detected in the blood (Sulzer et al., 2017; Lindestam Arlehamn et al., 2020) could have been activated in the brain (Figure 2), or in the periphery in draining lymph nodes (discussed below, Figure 3), but probably in a process taking place after neuronal dysfunction and microglia activation have occurred (Figures 2, 3). Alternatively, this could have happened in for instance the gut, as a consequence of an initial local inflammatory processes, but if and when gut pathology come into play in PD pathogenesis is not settled (discussed below; Figure 3). In the brain α-synuclein is exclusively produced by neurons (Jakes et al., 1994). Therefore, since microglia do not express α-synuclein, the antigen presentation to CD4+ T-cells in the brain must take place after microglia have phagocytosed extracellular α-synuclein (probably aberrant misfolded species; see Zhang et al., 2005; Lee et al., 2008). This implies that microglia get involved in the disease process as a consequence of an initial secretion of pathological α-synuclein from dysfunctional neurons (Figure 1). Thus, T-cells are probably activated downstream of microglia activation (Figure 2).

T-cell migration and infiltration into the brain parenchyma are tightly regulated at the level of the brain barriers (Nishihara et al., 2020). In spite of this, T-cell surveillance in the human brain does occur, not only in PD (Brochard et al., 2009; Sommer et al., 2018), but also in individuals without any known brain disease (Smolders et al., 2018). Thus, the possibility exists that patrolling CD4+ T-cells can be primed in the brain when facing pathologic a-synuclein peptides on microglial MHC class II (Figure 2). The invasion of CD8+ T-cells is in fact larger than that of CD4+ T-cells (Brochard et al., 2009; Smolders et al., 2018), indicating that also CD8+ cells can be initially primed in the brain (Figure 2). Interestingly, it has been shown that dopamine neurons in the SN of PD patients express MHC class I (Cebrián et al., 2014). In this way endogenous aberrant α-synuclein in dopamine neurons could be presented via MHC class I to CD8+ T-cells (Figure 2). Moreover, using *in vitro* models Cebrián et al. (2014) showed that the expression of MHC class I on dopamine neurons was induced by production of INF-γ by activated microglia, thus putting also the possible brain priming of CD8+ T-cells downstream of microglia activation (Figure 2). Although T-cells are present in the PD brain, a prominent lymphocytic infiltrate, like that found in multiple sclerosis, is not observed in PD (cf. Brochard et al., 2009; Machado-Santos et al., 2018).

As the circulation of T-cells through the PD brain is probably limited, the T-cell priming event is more likely to take place outside the brain at specialized priming sites like peripheral lymph nodes (Figure 3). A scenario could be that pathological α-synuclein released from neurons into the brain interstitium drains with the glymphatic system (Iliff et al., 2012) to the CSF in the subarachnoidal space. From here pathological α-synuclein could reach cervical lymph nodes (Benner et al., 2008; Ahn et al., 2019) via the newly discovered meningeal lymph vessels (Aspelund et al., 2015; Louveau et al., 2015; Ahn et al., 2019). In the lymph nodes monocyte-derived dendritic cells may phagocytose these α-synuclein antigens and via MHC class II induce priming of CD4+ T-cells (Korn and Kallies, 2017). These primed T-cells can then infiltrate the brain. When facing antigen presenting microglia they may be reactivated starting a stronger local immune response (Figure 3). However, the profile and function of such activated CD4+ T-cells in the PD brain remain to be determined.

Although nothing is known about the repertoire of cytokines that is secreted by T-lymphocytes infiltrating the PD brain, peripheral T-lymphocytes from PD patients secrete for instance IFN-γ (Sulzer et al., 2017; Kustrimovic et al., 2018) and TNF-α (Kustrimovic et al., 2018). These cytokines have been shown to be involved in dopamine neurodegeneration in experimental PD models (Mount et al., 2007; Harms et al., 2013). Therefore, the situation in the PD brain (Figure 2) may be similar to that in the brain of patients with multiple sclerosis, in which infiltrating CD4+ T-cells secrete neurotoxic INF-γ and TNF-α (Fletcher et al., 2010). Moreover, INF-γ may increase the expression of MHC class II on microglia (Panek and Benveniste, 1995), and as mentioned above that of MHC class I on dopamine neurons. This will further enhance dopamine neuronal toxicity. INF-γ is probably secreted by CD4+ T-cells, but this could also be done by microglia (Kawanokuchi et al., 2006; Mount et al., 2007; Cebrián et al., 2014).

To sum up, what is known about adaptive immunity in the human PD brain is that microglia and dopamine neurons in the SN express MHC class II and MHC class I molecules, respectively. CD4+ and CD8+ T-cells are enriched in this brain region. The mechanisms whereby they contribute to degeneration of dopamine neurons is still not clarified, but some of their putative roles are depicted in Figures 2, 3.

### Can Systemic Inflammation Lead to Brain Inflammation and Neurodegeneration?

#### Accumulation of α-Synuclein in Gut Neurons and Spreading to the Brain

Emerging data have proposed that systemic inflammation with T-cell priming can occur in the gut as a key event in PD pathogenesis. According to the Braak hypothesis (Braak et al., 2003) α-synuclein pathology could start outside the brain and transmit centrally from for example the intestine via the vagus nerve and the parasympathetic dorsal motor nucleus of the vagus (Breen et al., 2019). There are indeed indications that α-synuclein pathology is present in peripheral organs, such as the intestine (Braak et al., 2006). Thus, it has been speculated that a pathological process, such as inflammation, in the gut could trigger pathologic α-synuclein aggregation in local neurons. This could make the gut the initiating site of inflammation, driving propagation of pathological α-synuclein to the brain in a prion-like fashion (see Kim et al., 2019 for experimental evidence), in turn leading to PD neuropathology and neuroinflammation as discussed above. This mechanism of neurodegeneration in PD brains could first and foremost depend on transsynaptic spread of pathological α-synuclein from the gut to the brain, i.e., it may not involve brain infiltration of T-cells (Figure 3).

Indeed, hyperphosphorylated α-synuclein (at S129) has been shown to be located in gut neurons before the start of motor symptoms in PD patients, but not in healthy controls (Shannon et al., 2012; Hilton et al., 2014). However, whether α-synuclein aggregation in the gut is unique for PD could be questioned, because also in healthy individuals phosphorylated α-synuclein in gut neurons has been observed (Böttner et al., 2012; Gray et al., 2014). In line with this, a recent meta-analysis estimated that the specificity for detecting α-synuclein in gut neurons in PD patients vs. controls was about 0.8 (Bu et al., 2019), meaning that 20% of controls displayed α-synuclein labeling of gut neurons. Moreover, when comparing the presence of α-synuclein in the gut and the brain in patients with incidental PD (regarded as a prodromal of PD), none of the cases showed α-synuclein only the gut and not the brain (Adler and Beach, 2016). This further questions whether the gut is an initial site of production of aberrant α-synuclein. The possibility exists that α-synuclein could be transported bidirectionally in the vagus nerve, indicating that aberrant α-synuclein found in gut neurons in PD patients could originate from the brain (Breen et al., 2019). Furthermore, α-synuclein has been detected in the vagus nerve (Beach et al., 2010) and two studies noted that vagotomy decreased the risk of PD (Svensson et al., 2015; Liu et al., 2017), but this was not found in another study (Tysnes et al., 2015). Neither the presence of α-synuclein in the vagus nerve nor the reported effect of vagotomy on PD risk can be taken as evidence of gut-to-brain spread, at least as an initial event. First, vagotomy could affect an initial vagal transport of α-synuclein from brain to gut neurons. This would thereby reduce a secondary triggering of gut T-cell responses that would otherwise have been transmitted to the brain, enhancing brain inflammation and neurodegeneration. Second, vagotomy disturbs the physiological regulation of the vagal nerve on peripheral inflammation (Matteoli et al., 2014), obscuring the α-synuclein relation to PD development.

Thus, there are uncertainties as to whether gut-to-brain transmission of pathological α-synuclein via the vagus nerve may start brain inflammation and neurodegeneration. However, as discussed above increased levels of pro-inflammatory cytokines in the blood of PD patients have been detected, suggesting that the parkinsonian immune system has responded to tissue damage and/or foreign or altered molecules, such as pathological α-synuclein. Moreover, the blood levels of cytokines correlate with the clinical stage of the disease, emphasizing a role for peripheral inflammation in PD progression (Reale et al., 2009). Interestingly, there is evidence for local inflammation in the gut in patients with PD. Increased levels of the pro-inflammatory cytokines TNF-α, IL-1β, IL-6, and IFN-γ have been found in colon biopsies in PD compared to controls (Devos et al., 2013). The cytokine levels were negatively correlated with disease duration, suggesting that bowel inflammation is an early event in PD. Supporting this is the finding that aggregation of α-synuclein in gut neurons is associated with increased intestinal leakiness to pro-inflammatory bacterial products in patients newly diagnosed with PD (Forsyth et al., 2011). Such tissue pathology and inflammatory changes could lead to activation of T-cells in the gut. Indeed, CD4+ T cells are present in the colon of patients with PD and more so in patients with constipation than in those without constipation (Chen et al., 2015). The above mentioned findings show that the gut is subjected to inflammation and α-synuclein aggregation in PD, but whether this is an initial event triggering PD pathogenesis and its role in disease progression remains to be proven (Figure 3).

#### Evidence From Pharmaco-Epidemiological Studies

The question is if data from studies on the inflammatory bowel diseases (IBD) ulcerative colitis and Crohn’s disease can be used to throw light on PD pathogenesis. Data from such studies can contribute in two ways: They can clarify (1) the association between gut inflammation and PD, and (2) indirectly the role of T-cells in PD; if primed T-cells play an active role in neurodegeneration one should expect that peripheral acting immunosuppressant agents used in the treatment of IBD will slow or halt PD progression. Concerning the first question, three studies have noted that patients with IBD show increased likelihood of developing PD (about 20–30% increased risk of PD as compared with non-IBD individuals; Peter et al., 2018; Villumsen et al., 2019; Weimers et al., 2019). On the other hand, one study showed the opposite, i.e., reduced risk of PD (about 15% reduced risk of PD as compared with non-IBD individuals; Camacho-Soto et al., 2018). When it comes to the second question, certain types of immunosuppressant have been shown to give reduced risk of developing PD. One study observed this for corticosteroids and inosine monophosphate dehydrogenase (IMDH) inhibitors (e.g., azathioprine), but not for anti-TNF-α therapy (Racette et al., 2018). In contrast, Peter et al. (2018) concluded that TNF-α blockade was associated with reduced risk of PD. In Racette et al. (2018) they included patients newly diagnosed with PD and a random selection of controls without PD. The authors identified how many persons in the two groups who were prescribed with some of seven types of immunosuppressant until a year before inclusion in the study. Only corticosteroids and IMDH inhibitors showed a potential to reduce the risk of PD, with the latter drug being the most effective (35% reduction in PD incidence). This is interesting because IMDH inhibitors block DNA synthesis preferentially in T-lymphocytes (Thomas et al., 2005), and as this class of drugs does not cross the blood brain barrier, they may work through dampening peripheral T-cell mediated inflammation. The study by Peter et al. (2018) noted that there was a decreased incidence of PD among the inflammatory bowel disease patients who took anti-TNF-α therapy. Despite this effect there was an increase in PD risk in the entire IBD population, where the risk of ulcerative colitis was similar to that of Crohn’s disease. The latter result is in conflict with data in Villumsen et al. (2019) and Weimers et al. (2019), where the PD risk was significantly higher among patients with ulcerative colitis compared to those with Crohn’s disease. In addition, in the Swedish study (Weimers et al., 2019) the effect disappeared when the authors adjusted for the numbers of medical visits. Although these epidemiological/pharmacoepidemiological studies indicate that there is a possible association between inflammation in the gut and development of PD and that certain types of immunosuppressants may reduce the incidence of PD, there are clear discrepancies. These cast uncertainty on the results.

It should also be mentioned that non-steroid anti-inflammatory drugs (NSAIDs) have been extensively examined for neuroprotective effects. Experimental PD models have shown somewhat confliction results. Acetyl salisylate and ibuprofen have been reported to be neuroprotective in MPTP mouse models of PD (Aubin et al., 1998; Swiątkiewicz et al., 2013). However, using the same model, recently ibuprofen given alone was not protective (only when given along with a metalloprotease inhibitor) (Costa et al., 2020). Likewise, results from epidemiological studies are somewhat conflicting. Two meta-analyses concluded that ibuprofen could slightly lower the risk of PD (Samii et al., 2009; Gagne and Power, 2010). However, later this has not been verified in meta-analyses including a larger number of studies (Ren et al., 2018; Poly et al., 2019). There are no randomized clinical trials investigating the effect of NSAIDs. Likewise, rosacea patients who have used tetracyclines appear to have a reduced risk of PD (Egeberg et al., 2016). As mentioned above, tetracyclines may work to reduce inflammation, but in clinical trials using minocycline there was no effect on PD progression (NINDS Net-PD Investigators, 2008; Parashos et al., 2014).

### Clinical Randomized Trials Investigating the Effect of Anti-inflammatory Agents

The only way to answer the question about whether targeting inflammation can rescue dopamine neurons from dying in PD patients is to conduct proper randomized clinical trials. As mentioned above, several clinical trials testing anti-inflammatory drugs have been performed, but none have shown any significant neuroprotective effect (NINDS Net-PD Investigators, 2008; Parashos et al., 2014; NINDS Exploratory Trials in Parkinson Disease (NET-PD) FS-ZONE Investigators, 2015). This may be related to the trial design or to the possibility that the drugs in question were not potent enough or simply that targeting inflammation is not sufficient to change the course of PD. Of note is that one recent trial showed promising results using the glucagon-like peptide 1 receptor (GLP1R) agonist exenatide (Athauda et al., 2017). The authors reported that the drug improved motor function in PD patients compared to those given placebo. This is interesting because in a recent experimental study using two different PD models another type of GLP1R agonist (NLY01) was shown to have anti-inflammatory effects in the brain (Yun et al., 2018). By stimulating GLP-1Rs on microglia NLY01 prevented dopamine neuronal death in the PD animals. This happened in a cascade involving astrocytes as effector toxic cells (Yun et al., 2018). However, in the clinical PD setting it cannot be concluded that GLP1R agonists have neuroprotective effects, because there are several uncertainties and shortcomings with the exenatide study (Athauda et al., 2017): (1) It could not be determined if the effect was due to a symptomatic effect or if it represented neuroprotection. The drug effect was evident after quite a short time (12 weeks) and did not increase thereafter (48–60 weeks). (2) More importantly, the patients treated with exenatide had already reached a rather advanced disease stage with an average disease duration of 6.5 years. This is a stage where one should expect little or no effect of neuroprotective strategies (see discussion above). (3) The number of included PD patients was very low (30 in the exenatide group/30 in the placebo group). These numbers were based on a power analysis prior to the start of the study. In this calculation the authors used a difference of 5.8 points in the motor part of the Movement Disorders Society Unified Parkinson’s Disease Rating Score (MDS-UPDRS) between the treatment and placebo group. However, this is far too high a score, because motor symptoms in PD progress with about 1.5 point per year (Post et al., 2011; Lilleeng et al., 2014). This means that the maximum expected difference to be observed between the GLP1R agonist and placebo would be about 1.5 MDS-UPDRS points during the duration of the study (48 weeks). Altogether, these factors make it difficult to conclude about neuroprotection. In future trials newly diagnosed PD patients should be enrolled instead of patients with a marked disease progression. It is pivotal to study patients at the earliest possible stage, when there is a greater chance that the patients have vital dopamine neurons, which could benefit from the therapy. Next, the number of PD patients should be high enough to give a sufficient power to detect statistical differences. Given a study duration of 2 years, a sample size of about 250 patients is needed to detect a difference of 3.0 MDS-UPDRS points between two groups (treatment and placebo), assuming SD of 9 MD-UPDRS points (Post et al., 2011; Lilleeng et al., 2014) and a significance level of 5% (two-sided).

## Conclusion

There is ample evidence from human PD studies that microglia are activated early in the disease process and in any case within 3 years from diagnosis. It could be that microglia activation is among the initial inflammatory events that takes place in PD. This means that starting treatment with inhibitors of microglia activation at an early PD stage may be effective in slowing down or stop further neurodegeneration. It would be exciting to see data from studies further testing the effect of GLPR1 agonists. A phase II study using NLY01 is now recruiting (NCT04154072; clinicaltrials.com). This study is promising, because it will include a high number (*n* = 240) of newly diagnosed PD patients. However, the study duration is only 36 weeks, which may be too short to detect any neuroprotective effect (because the clinical worsening will be very small in the course of 36 weeks). This could be handled by an open extension of the trial. Despite that the knowledge about the significance of adaptive immunity and T-cell responses for PD pathogenesis is still rather limited, another option could be to block the access of T-lymphocytes to the PD brain. This may be achieved by repurposing drugs known to be disease modifying in multiple sclerosis. Along this line, testing also drugs that can broadly dampen peripheral inflammation, such as anti-TNF-α drugs or IMDH inhibitors may hold a promise. But as these drugs have some serious side effects there are ethical concerns with such studies. Whether Tregs play a role in the degeneration of dopamine neurons is also largely unknown. But from the discussion above it could be a rationale for testing agents that may increase the level of functional Tregs and supress production of autoreactive T-cells. Such a drug could be glatiramer acetate, which is approved for treatment of multiple sclerosis (Prod’homme and Zamvil, 2019). At last, the important question is if inflammation plays a sufficient pathogenetic role in neurodegeneration that interfering with it will significantly alter disease progression. So far there are no human studies directly showing this. But along with data suggesting that inflammation is present in the brains of PD patients from an early disease stage, and even at prodromal stages, a wealth of data from experimental PD models show neuroprotection of anti-inflammatory treatments. This strongly suggests that inflammatory cascades do have an important impact on development of neurodegeneration in PD. This justifies initiating more clinical trials on inflammation in PD.

## Author Contributions

VG wrote the manuscript.

## Conflict of Interest

The authors declare that the research was conducted in the absence of any commercial or financial relationships that could be construed as a potential conflict of interest.
